# Molecular Mechanism of Inhibition of the Mitochondrial Carnitine/Acylcarnitine Transporter by Omeprazole Revealed by Proteoliposome Assay, Mutagenesis and Bioinformatics

**DOI:** 10.1371/journal.pone.0082286

**Published:** 2013-12-09

**Authors:** Annamaria Tonazzi, Ivano Eberini, Cesare Indiveri

**Affiliations:** 1 CNR Institute of Biomembranes and Bioenergetics, Bari, Italy; 2 Laboratorio di Biochimica e Biofisica Computazionale, Dipartimento di Scienze Farmacologiche e Biomolecolari, Sezione di Biochimica, Biofisica, Fisiologia ed Immunopatologia, Università degli Studi di Milano, Milano, Italy; 3 Department BEST (Biologia, Ecologia, Scienze della Terra), Unit of Biochemistry and Molecular Biotechnology, University of Calabria, Arcavacata di Rende, Italy; University of Salento, Italy

## Abstract

The effect of omeprazole on the mitochondrial carnitine/acylcarnitine transporter has been studied in proteoliposomes. Externally added omeprazole inhibited the carnitine/carnitine antiport catalysed by the transporter. The inhibition was partially reversed by DTE indicating that it was caused by the covalent reaction of omeprazole with Cys residue(s). Inhibition of the C-less mutant transporter indicated also the occurrence of an alternative non-covalent mechanism. The IC_50_ of the inhibition of the WT and the C-less CACT by omeprazole were 5.4 µM and 29 µM, respectively. Inhibition kinetics showed non competitive inhibition of the WT and competitive inhibition of the C-less. The presence of carnitine or acylcarnitines during the incubation of the proteoliposomes with omeprazole increased the inhibition. Using site-directed Cys mutants it was demonstrated that C283 and C136 were essential for covalent inhibition. Molecular docking of omeprazole with CACT indicated the formation of both covalent interactions with C136 and C283 and non-covalent interactions in agreement with the experimental data.

## Introduction

Mitochondria represent important targets of pharmacological compounds. Indeed, exogenous substances interacting with mitochondrial enzymes or transporters may influence the entire cell metabolism. Among many proteins which have been shown to interact with pharmacological compounds [1], the carnitine/acylcarnitine transporter (CACT) emerged as a target of drugs [2], [3]. The CACT has an essential role in the β-oxidation of fatty acids [4]. From studies performed in intact mitochondria and in proteoliposomes reconstituted with the purified or the recombinant protein, the function of the transporter has been well assessed. Physiologically, the transporter allows the entry of acyl groups, as acylcarnitines, into the mitochondrial matrix, for the β-oxidation. The free carnitine which is released in the matrix from acylcarnitine is transported back to the cytosol by the same transporter which catalyses an antiport reaction [4], [5]. Inherited defects of the CACT gene cause a syndrome which is known as secondary carnitine deficiency [6] and is more severe than the primary carnitine deficiency caused by defects of the plasma membrane transporter OCTN2 [7]. The structure/function relationships of the transporter have been well characterized and several properties have been clarified using combined experimental approaches based on site-directed mutagenesis, chemical targeting, functional analysis in proteoliposomes and bioinformatics [8]–[11]. The transporter is functionally and structurally asymmetrical and it is inserted in the proteoliposomal membrane in a right-side out orientation as compared to mitochondria [12]. Thus the proteoliposome system is suitable for obtaining physiologically relevant data, in absence of interferences by other transporters or enzyme pathways. The CACT possesses 6 Cys residues whose roles in the protein function have been defined [9], [13]. One of these residues, C136, is located in the middle of the central cavity of the transporter and is responsible for the reactivity with SH reagents, such as N-ethylmaleimide (NEM) which inactivate the CACT. C155 can form a disulphide with C136 since, during some steps of the catalytic cycle, it comes close to C136. C23 should be involved in the interaction with the membrane, while C58 should interact with cardiolipin. C89 and C283 seem to be less important for the protein function, even though these residues are exposed in the upper level of the central cavity. Due to the presence of several Cys residues, the CACT may interact with pharmacological compounds exhibiting reactivity towards thiol groups. Among the compounds with this chemical property, omeprazole, has been well described in terms of mechanism of pharmacological action. It reduces gastric secretion acting on the K^+^/H^+^-ATPase [14]–[18]. The pharmacological agent is activated at acidic pH in the gastric lumen and, after chemical modifications, it becomes reactive towards an SH group of the K^+^/H^+^-ATPase forming a mixed disulphide, which is responsible of the inhibition of the proton pump. A similar mechanism of interaction of omeprazole was also described for the plasma membrane carnitine (OCTN2) transport system of rat kidney. The drug causes inactivation of the transporter, by forming mixed disulphides with Cys residues of the protein [19]. By analogy, the mitochondrial carnitine/acylcarnitine transporter (CACT) may be a further potential target of omeprazole. Interestingly it has been found that omeprazole strongly inhibits the transport system. The structure/function relationships and the mechanism of the interaction of omeprazole with the CACT have been characterized by site-directed mutagenesis combined with molecular docking.

## Materials and Methods

### Materials

Sephadexes G-50, G-75 and G-200 were purchased from Pharmacia, l-[methyl-^3^H]carnitine 99% pure, 85 Ci/mmol, from Scopus Research BV, Wageningen The Netherlands, egg-yolk phospholipids (l-α-phosphatidylcholine from fresh turkey egg yolk), 1,4-piperazinediethanesulfonic acid (Pipes), Triton X-100, cardiolipin, l-carnitine and N-dodecanoylsarcosine (sarkosyl) from Sigma-Aldrich. All other reagents were of analytical grade.

### Site-directed mutagenesis, overexpression and isolation of the CACT proteins

The previously constructed pMW7-WTratCACT recombinant plasmid containing the wild-type (WT) CACT cDNA was used to introduce the mutations in the CACT protein [12]. The amino acid replacements were performed with complementary mutagenic primers using the overlap extension method and the High Fidelity PCR System (Roche). The PCR products were purified and digested with NdeI and HindIII (restriction sites added at the 5′ end of forward and reverse primers, respectively) and ligated into the pMW7 expression vector. All mutations were verified by DNA sequencing, and, except for the desired base changes, all the sequences were identical to that of rat CACT cDNA. The resulting plasmids were transformed into *E. coli* C0214. Bacterial overexpression, isolation of the inclusion body fraction, solubilization and purification of the wild-type CACT and mutant CACT proteins were performed as described previously [12], [20].

### Reconstitution of wild-type and mutant CACT proteins in liposomes

The recombinant proteins were reconstituted into liposomes as described previously [12], [20]. Briefly, a mixture was prepared with 60 µg protein, 1% Triton X-100, 10 mg phospholipids in the form of sonicated liposomes, 10 mM Pipes pH 7.0 and 15 mM carnitine in a final volume of 700 µl. The mixture was passed 15 times through the same Amberlite column (0.5 cm diameter x 3.2 cm height). The external substrate was then removed from proteoliposomes on Sephadex G-75 columns before the transport assay.

### Transport measurements

Transport at 25°C was started by adding 0.1 mM [^3^H]carnitine to proteoliposomes and terminated by the addition of 1.5 mM NEM. In controls, the inhibitor was added together with the labeled substrate, according to the inhibitor stop method [21], [22]. This strategy allows subtracting, from the experimental samples, the aliquot of radiolabelled carnitine diffusing through the liposomal membrane. Finally, the external substrate was removed by chromatography on Sephadex G-50 columns, and the radioactivity in the liposomes was measured. The experimental values were corrected by subtracting control values. For efflux measurements, the proteoliposomes were loaded with radioactivity before starting the transport assay. This was achieved by incubating the proteoliposomes (600 µl), passed through Sephadex G-75 as described in Materials and methods, with 5 µM [^3^H]carnitine with a specific radioactivity of 10 nCi/nmol, for 60 min at 25°C. Then, the external radioactivity was removed by passing again the proteoliposomes through Sephadex G-75 as described above except that this chromatography was performed at 4°C to minimize the loss of internal substrate during the chromatography. Transport (efflux) was started by adding external buffer or substrate and stopped at the appropriate time interval. The transport assay temperature was 25°C. Finally, each sample of proteoliposomes (100 µl) was passed through a Sephadex G-50 column to separate the external from the internal radioactivity. Efflux activity was expressed as intraliposomal cpm. Activation of omeprazole was obtained as follows: 25 mM omeprazole in ethanol was diluted 10 fold in HCl 0.1 M and incubated for 15 min. Then, the pH was adjusted to pH 6.0 by Tris powder.

### Molecular modeling, docking, and low-mode molecular dynamics

CACT molecular model was built through the Swiss Model server (http://swissmodel.expasy.org) after an alignment with the bovine ADP/ATP carrier sequence. The alignment was inspected and manually adjusted with the Deep View software [23], and the output was used to run a SwissModel procedure in ‘Project Mode’.

Molecular docking was carried out with the Molecular Operating Environment (MOE) 2011.10. A database of the activated forms for protonic pump inhibitors (omeprazole, pantoprazole and lansoprazole) was built through the Builder module of MOE 2011.10. The structures were energy minimized with the MMFF94x force field. The final structures were saved in a database (mdb) file and then used for the molecular docking simulations.

The CACT binding site was identified through the MOE Site Finder module. This method is purely geometric and based not on energy models but on alpha spheres, which are a generalization of convex hulls [24]. Dummy atoms were positioned inside the binding site in order to carry out a more focused molecular docking procedure.

Docking was carried out with the MOE Dock program in its standard ‘Rigid Receptor’ mode (with all the parameters set as default). For each of the inhibitors bound to CACT, the top scoring solutions, as ranked according to the GBVI/WSA dG function, were selected. In order to optimize the interactions between ligands and receptor and to compute the affinity through the London dG empirical scoring function they were then submitted to a further refinement through the MOE LigX module. The dissociation constants for the complexes, expressed as pKi (-Log Ki), were computed from the affinity data.

Low mode molecular dynamics (LowModeMD) was used to generate putative conformations of CACT C89, C136, and C238 covalently bound to activated omeprazole. LowModeMD is aimed at focusing a MD trajectory along the low-mode vibrations and features a very efficient way versus classical MD for searching for minima troughs on the potential energy surface. To run these computations, we used the MOE Conformational Search program. This program uses an efficient implicit method for estimating the low-frequency modes and is based on the attenuation of high-range velocities, as described in detail in [25]. A molecule of activated omeprazole was bound to C89 or C136 or C238 in CACT with the MOE Builder. The omeprazole atoms were left free to move during the LowModeMD, whereas the residues more than 4.5 Å apart were fixed (not free to move, but used for the energy calculations); the other residues were defined as inert (fixed and not used for energy calculations). The simulation was carried out with default parameters. Two hundred and fifty structures were generated and ranked according to the value of the potential energy of the conformation. The conformations with strain energy lower than 2 kcal/mol versus the lowest energy conformation were analyzed. One among the conformations in which Cys238 was covalently bound to activated omeprazole (conformation #5) was used to dock omeprazole, according to the already described procedure.

### Other methods

SDS-PAGE was performed as previously described [12]. The amount of recombinant protein was estimated on Coomassie-blue stained SDS-PAGE gels by using the Chemidoc imaging system equipped with Quantity One software (Bio-Rad) as previously described [26].

## Results

### Inhibition of the carnitine transporter by omeprazole

**Figure 1:**
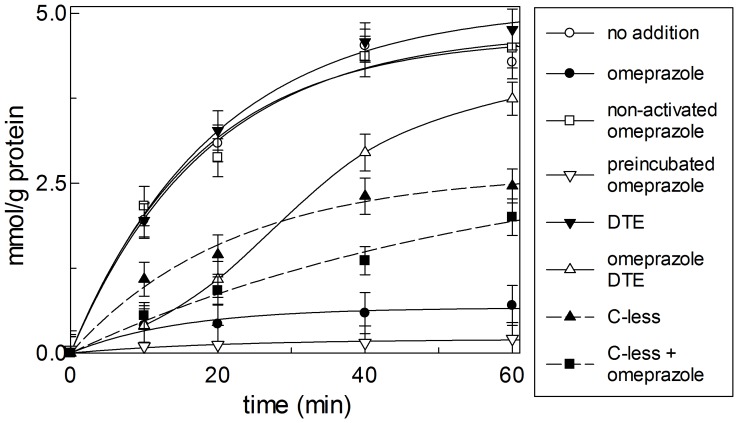
Effect of omeprazole on the carnitine antiport mediated by CACT. Transport was measured as described in Materials and methods adding 0.1[^3^H]carnitine at time zero to proteoliposomes containing 15 mM carnitine in the absence (○,▾,▴) or in the presence of activated (•, ▪) or non-activated (□) 30 µM omeprazole. In (▾) 2 mM DTE was added 2 min before the transport start. In (▵) 2 mM DTE was added 10 min after the start to the proteoliposomes treated with omeprazole. In (▿) the proteoliposomes were pre-incubated with omeprazole and passed through Sephadex G-75 column to remove the non reacted reagent before the transport assay. The transport reaction was stopped at the indicated times, as described in Materials and methods. Reported values are means ± S.D. from three experiments of mmol of transported substrate per g of protein (mmol · g protein^−1^).

The effect of omeprazole on the carnitine transporter reconstituted in liposomes was investigated. The transport activity was measured as 100 µM [^3^H]carnitine uptake into proteoliposomes containing 15 mM carnitine, (carnitine/carnitine homologous antiport) in the presence of externally added 30 µM omeprazole activated by pre-incubation at pH 1.0 (see Materials and methods and refs. [15]–[18]), or non activated, i.e. kept in a buffer at pH 7.0 before the use. The time course of the carnitine transport is shown in Fig. 1. The accumulation of labeled carnitine into the proteoliposomes depended on the time, it increased up to 4.2 mmol g protein^−1^ at 60 min. 30 µM activated omeprazole strongly inhibited the transport. At 10 min about 80% inhibition was found. At 60 min the transport was still inhibited by about 85%; whereas the addition of the non-activated compound had no inhibitory effect. The experimental data were interpolated in a first order rate equation from which the initial rate of transport was calculated as the product of the first order rate constant, k and the transport at equilibrium. The initial transport rate in the presence or absence of omeprazole was 0.30 or 0.045 mmol min^−1^ g protein^−1^, respectively. The two forms of activated omeprazole have functional groups [17] that could react with Cys residues of the protein forming a mixed disulphide, as it was previously found in the case of the gastric H^+^/K^+^-ATPase [16], [17] and of the plasma membrane OCTN2 transporter [19]. SH group(s) of Cys of the CACT are exposed towards the external side of the proteoliposomes, which corresponds to the cytoplasmic side of the transporter [9], [27]. These Cys could be involved in the mechanism of inhibition by omeprazole via the formation of mixed disulphide(s). To evaluate this hypothesis the effect of dithioerythritol (DTE), a disulphide reducing agent, was tested. The addition of DTE to the proteoliposomes, after 10 min treatment with activated omeprazole, led to partial recovery of the transport function, which was clearly evident at 60 min of transport: about 78% of the activity was rescued respect to the inhibited sample. As a control, DTE was added to the untreated protein; no appreciable effects were observed. The incomplete recovery of the transport function caused by DTE may indicate that only a fraction of the transporter-omeprazole mixed disulphide linkage was reversed by DTE or that omeprazole was also able to inhibit the transporter by interactions different from the disulphide formation. To ascertain the presence of non-covalent interaction, the same experiment was performed on the C-less CACT, which has an intrinsic activity lower than the WT [27] but sufficient for evaluating the effect of the inhibitor. Indeed, omeprazole inhibited also the C-less protein, even though at a lower extent respect to the WT with a calculated initial rate of 0.051 mmol min^−1^ g protein^−1^, i.e. 40% of that of C-less CACT in the absence of omeprazole, 0.127 mmol min^−1^ g protein^−1^ (Fig. 1). No recovery of transport function was exerted by the addition of DTE in the case of the C-less CACT (not shown). This data indicated that the compound interacted with the protein also in the absence of Cys residues, i.e. non-covalently. To unequivocally demonstrate that the inhibition observed on the WT protein was actually due to the formation of mixed disulphide(s) between omeprazole and SH residues of the protein, a strategy, previously described for other transporters [3], [19], [28], was adopted. The proteoliposomes reconstituted with the wild type CACT were pre-incubated with omeprazole and then passed through Sephadex G-75 columns to remove the excess reagent. As shown in Fig. 1, the inhibition was maintained after the Sephadex G-75 chromatography, indicating that omeprazole was covalently bound (by mixed disulphide) to the transporter. Since the formation of mixed disulphides should be facilitated by alkaline pH, a similar experiment was performed at pH 8.0, even though at this pH the CACT has a lower activity. The extent of inhibition was nearly identical than that at pH 7.0 (not shown).

### Kinetics of the inhibition of the carnitine transporter by omeprazole

**Figure 2:**
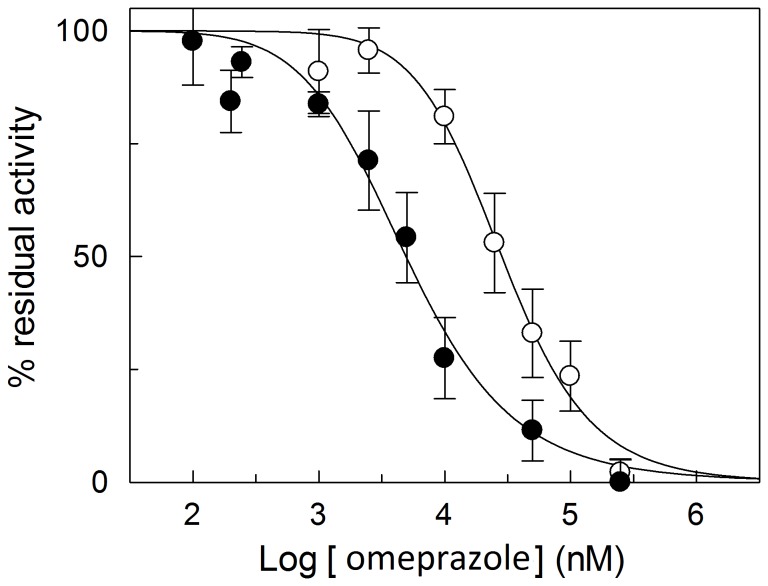
Dose-response curves for the inhibition of the reconstituted WT and C-less CACT by omeprazole. The carnitine/carnitine antiport rate was measured as described in Materials and methods, adding 0.1 mM [^3^H]carnitine together with the indicated concentrations of omeprazole to proteoliposomes reconstituted with WT (•) or C-less (○) CACT. % residual activities with respect to the control are reported. The values are means ± S.D. from three experiments.

**Figure 3:**
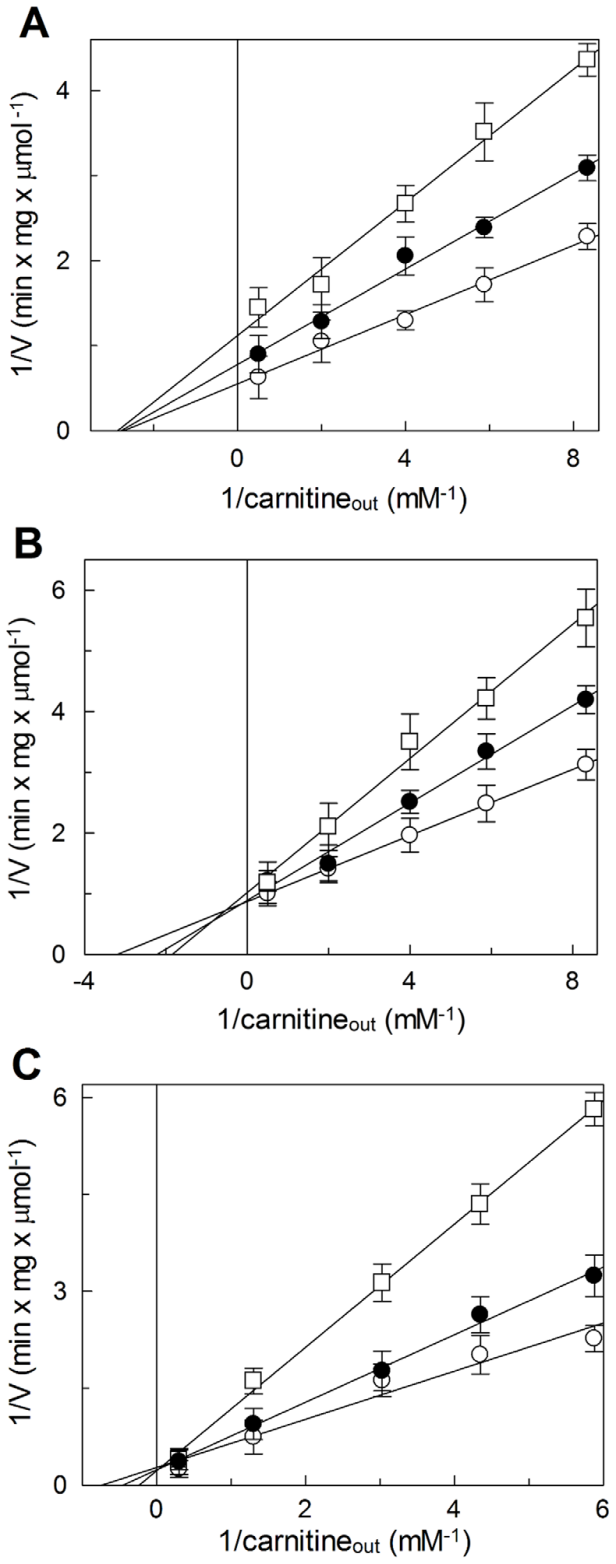
Kinetic analysis of the inhibition of the reconstituted CACT by omeprazole. The carnitine/carnitine antiport rate was measured, as described in Materials and methods, adding [^3^H]carnitine at different concentrations to proteoliposomes reconstituted with WT CACT (A–C) or C-less CACT (B) containing 15 mM carnitine, in the absence (○) or in the presence of external omeprazole to proteoliposomes. In A, B and C the respective omeprazole concentrations were: 5-15–20 µM (•) and 10–40–68 µM (□). In C, 2 mM DTE was added together with omeprazole. Experimental data plotted according to Lineweaver-Burk as reciprocal transport rate vs reciprocal carnitine concentrations. Reported values are means ± S.D. from three experiments of reciprocal of µmol of transported substrate per mg of protein per min (min · mg · µmol^−1^).

**Figure 4:**
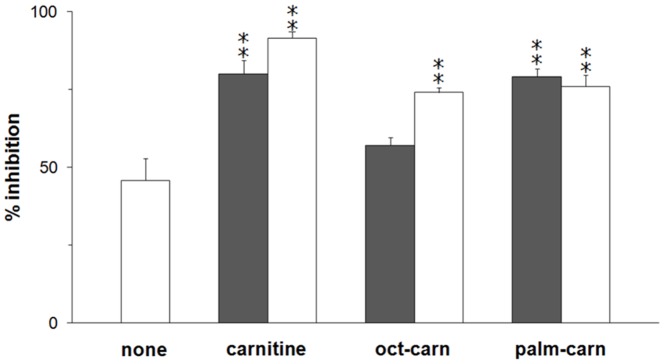
Effect of substrates on CACT inhibition by omeprazole. Carnitine (1 mM grey;15 mM white column), octanoyl-carnitine (oct-carn: 0.05 mM grey; 0.5 mM white column) or palmitoyl-carnitine (palm-carn: 0.02 mM grey; 0.1 mM white column) were added together with omeprazole to proteoliposomes reconstituted with the WT CACT and previously passed onto a Sephadex G75 column to remove the external carnitine. After 5 min incubation un-reacted compounds were removed by passing the proteoliposomes through Sephadex G-75 as described in Materials and methods. Transport was started adding 0.1 mM [^3^H]carnitine and stopped after 30 min. Percent residual activity is reported with respect to the control without the addition of omeprazole. The data represent means ± S.D. of three independent experiments. Significantly different from the sample treated with omeprazole (white column-none) in the absence of substrates, as estimated by the Student's t test (**p<0.01).

To characterize the effect of omeprazole caused by the two different mechanisms of interaction with the transporter, kinetic experiments were performed on the WT or C-less protein. The dependence of the inhibition on the concentration of omeprazole was studied. The antiport rate, measured as uptake of 100 µM [^3^H]carnitine into proteoliposomes containing 15 mM carnitine, was determined in the presence of increasing concentration of the pharmacological agent. The dose response curves obtained are shown in Fig. 2. Nearly complete inhibition of the transport was observed with the WT at about 300 µM omeprazole; the calculated IC_50_ from 3 experiments was 5.4±0.23 µM. In the case of C-less protein maximal inhibition was observed at 1.8 mM and the IC_50_ was 29±8.5 µM. The same experiment was performed on the WT, adding omeprazole to the proteoliposomes in the presence of DTE. Under this condition i.e., the pharmacological agent could not covalently bind the transporter (see Materials and methods), the experimental data resembled those obtained with the C-less protein (not shown). The IC50 of the WT CACT were also determined for pantoprazole (6.4±0.33 µM) and lansoprazole (80±23 µM) (experiments not shown). Inhibition kinetic studies were further performed on omeprazole to obtain data on the localization of the omeprazole binding respect to the substrate binding site. The dependence of the antiport rate on the external carnitine concentration was studied in the absence or presence of activated omeprazole. The data obtained on the WT and C-less CACT, analyzed in double reciprocal (Lineweaver-Burk) plot are shown in Fig. 3. In the case of WT (Fig. 3 A) the experimental data were interpolated by three straight lines, which showed a common intersection point on the abscissa. This behavior is typical of non-competitive inhibition. The half saturation constant (Ki) for the inhibitor, derived from the plot, was 10.6±1.6 µM (from three experiments). In the case of the C-less protein (Fig. 3B), the data were interpolated by three straight lines that, differently from the WT experiment, showed a common intersection close to the ordinate, typical of a competitive type of inhibition. The half saturation constant (Ki) for the inhibitor, derived from the plot, was 29.5±1.2 µM, higher than that obtained for the WT. Results similar to those obtained with the C-less protein were observed on the WT in the presence of DTE, with a Ki of 42.5±14.9 µM (Fig. 3 C). In the case of covalent inhibition, as that determined by omeprazole on the WT, a non-competitive pattern could be obtained also if the inhibition site is in the substrate binding site. This can be discriminated studying the effect of protection of substrate on the inhibition as described in Fig. 4. The incubation of the proteoliposomes with 30 µM omeprazole in the absence of carnitine led to inhibition of 46±7%. If 1 mM or 15 mM carnitine was added before the omeprazole, surprisingly, the inhibition increased to 80% and 92%, indicating that the omeprazole reaction with the transporter was not protected but facilitated by the presence of carnitine. A similar experiment was performed with other substrates of the CACT which have higher affinity respect to carnitine, octanolylcarnitine (0.05–0.5 mM) and palmitoylcarnitine (0.02–0.1 mM). Also the pre-incubation with these substrates increased the inhibition extent to 57% and 74% in the case of octanolylcarnitine or to 79% and 76% in the case of palmitoylcarnitine, respectively.

### Identification of cysteine residues responsible for omeprazole covalent inhibition

**Figure 5:**
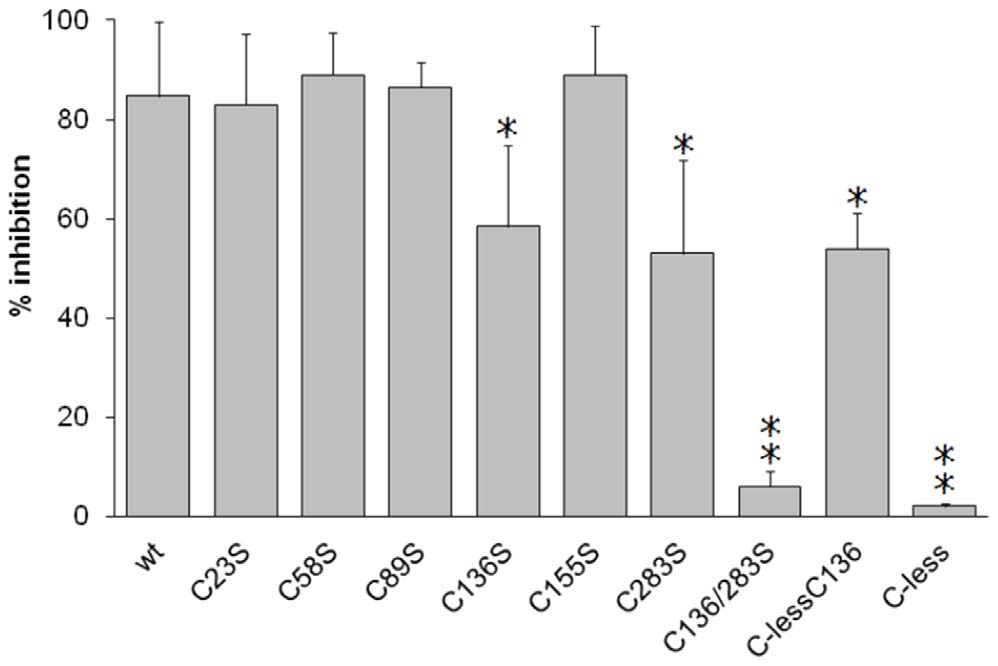
Inhibition of WT and Cys mutants of CACT. Transport rate was measured by adding 0.1 mM [^3^H]carnitine to proteoliposomes and stopped after 30 min. Omeprazole 30 µM was added to the proteoliposomes reconstituted with the various mutants and incubated for 5 min. The un-reacted compound was removed by passing the proteoliposomes through Sephadex G-75 as described in Materials and methods. The % inhibition with respect to the control are reported. In the legend the Cys residues substituted in the mutant proteins are indicated. The values are means ± S.D. from three experiments. Significantly different from the WT CACT inhibited by omeprazole (WT column), as estimated by the Student's t test (*p<0.05; **p<0.01).

To identify the Cys residue(s) that could react with omeprazole, the inhibition of Cys mutants of CACT lacking one or more Cys residues was analyzed. All the mutants lacking only one of the Cys residues were inhibited by more than 80% except the C136S, which was inhibited by 59% and the C283A, which showed an inhibition of 53% (Fig. 5). This indicated a major role of C136 and C283 in the interaction with omeprazole. The results were confirmed by the 54% inhibition of the mutant containing only C136 (C-less/V136C) and lack of inhibition of the double mutant C136/283S. As expected, also the C-less was not inhibited.

### Investigation of omeprazole transport

**Figure 6:**
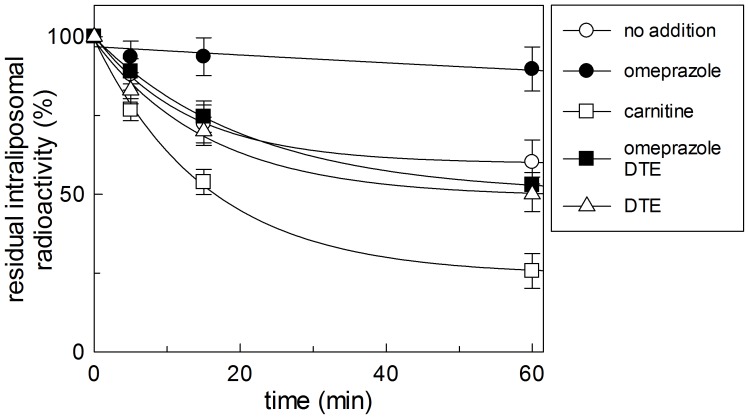
Effect of omeprazole on the carnitine efflux from proteoliposomes. Efflux of 15[^3^H]carnitine from pre-labeled proteoliposomes was measured as described in Materials and methods in the presence of only external 10 mM Pipes pH 7.0 (no addition), omeprazole 0.15 mM, 5 mM DTE and carnitine 0.5 mM according to the legend added at time 0. The values are means ± S.D. from three experiments.

According to the interaction of omeprazole with the substrate binding site, the CACT might mediate the transport of omeprazole. Since this transporter works by an antiport mode, the transport of the pharmacological agent can be evaluated using it as counter-substrate of [^3^H]carnitine. To this aim, proteoliposomes were loaded with [^3^H]carnitine by transporter-mediated exchange equilibration (see Materials and methods). After this procedure the proteoliposomes contained internal 15 mM [^3^H]carnitine. In the absence of external substrate some efflux of intraliposomal radioactivity was detected (Fig. 6), which was due to the unidirectional transport activity of the CACT [29]. In another set of experimental sample 0.15 mM carnitine was externally added. This addition caused stimulation of the efflux due to the induction of the antiport activity. The concentration of carnitine was kept relatively low (0.15 mM) to be comparable with that of omeprazole, the addition of which caused complete inactivation of the transport activity. The addition of omeprazole together with DTE, to prevent reaction with SH groups of the protein, led to efflux of the same amount of radioactivity as in the case of absence of external substrate. The data demonstrated that omeprazole could not be transported from outside to inside the proteoliposomes in exchange for internal [^3^H]carnitine.

### Molecular modeling

**Figure 7:**
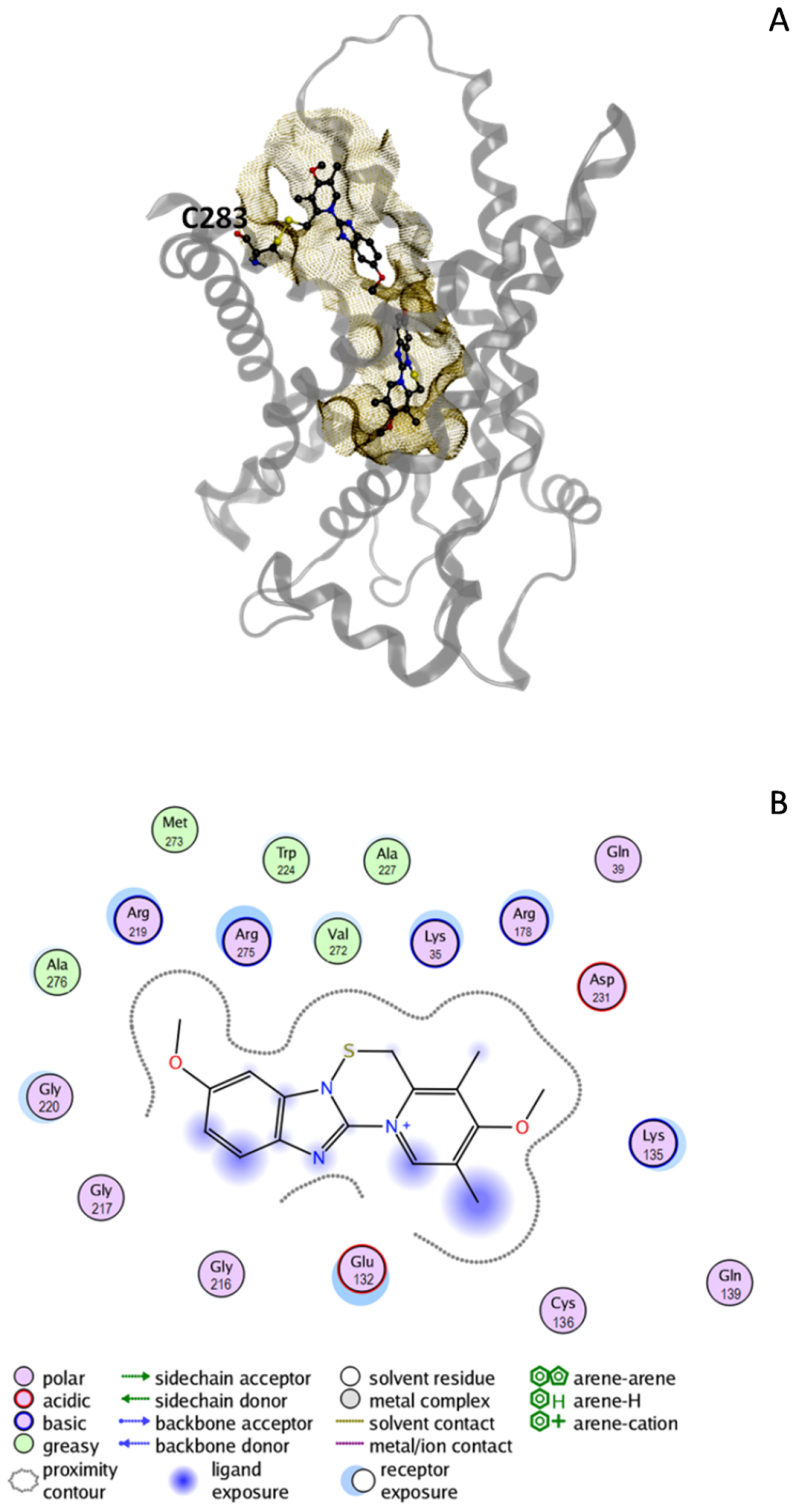
Interaction of omeprazole with CACT. (A) Ribbon representation of CACT with two bound molecules of omeprazole: one covalently bound to C283, as computed through LowModeMD; another non-covalently bound inside the CACT central cavity, as computed through molecular docking on the top-scoring covalent complex previously obtained thorugh LowModeMD. The molecular surface of the protein, close to the ligands, is dotted in yellow. (B) ligand interaction plot representing the CACT residues involved in omeprazole molecular recognition. The depicted complex is the top-scoring one as obtained through molecular docking. Associated docking score and computed binding free energy are reported in Table 1. A caption describing all the detected interactions is included in the picture.

Molecular docking of omeprazole and also of pantoprazole and lansoprazole demonstrated that these ligands can form stable complexes with CACT through a set of non-covalent interactions. The docking score was used to select the top scoring poses among the 30 generated for each ligand. All the values were negative, suggesting that these ligands can bind to CACT central cavity. The computed dissociation constants (K_i_) for the top scoring complexes suggest the following rank of affinity towards CACT: omeprazole ≃ pantoprazole > lansoprazole (Tab. 1). For omeprazole, which has been extensively characterized in the reported experiments, we computed a K_i_ of approx. 22 µM. In Figure 7B, a plot showing the CACT amino acids involved in omeprazole molecular recognition are highlighted. Several basic residues (K35, K135, R178, R219 and R275), two acidic residues (E132 and D231), a cysteine (C136), and both some polar and some apolar residues are clearly identified as interacting with omeprazole. Very interestingly most of these residues were previously found to be involved in the binding of substrate [30]

**Table 1:** Docking scores (kcal/mol) and binding free energies (kcal/mol) for the top-scoring complexes between CACT and three protonic pump inhibitors.

Ligand	Docking score (kcal/mol)	Binding free energy (kcal/mol)	Calculated Ki (µM)	pKi
Omeprazole	−6.49	−6.31	22	4.64
Pantoprazole	−6.46	−6.36	21	4.68
Lansoprazole	−5.45	−5.41	104	3.98

Three LowModeMD simulations were performed in order to evaluate the possibility that activated omeprazole could covalently bind to CACT cysteines, C89, C136 and C238. We analyzed 250 solutions for each simulation, and counted the number of solutions with strain energy lower than 2 kcal/mol versus the conformation with the lowest energy. We also evaluated the interaction potential energies between omeprazole and CACT of the lowest energy solutions for the three simulations (one for each of the studied cysteines). From our computations, C283 seems the most likely to covalently bind to omeprazole, since 9 conformations out of 250 showed a strain energy lower than 2 kcal/mol and the best solution had an energy of −29.9 kcal/mol. An intermediate reactivity can be hypothesized for C89, with 3 conformations under 2 kcal/mol and potential omeprazole-CACT interaction energy of −23.6 kcal/mol. The least reactive should be C136, with just 2 solutions under 2 kcal/mol and potential omeprazole-CACT interaction energy of −19.3 kcal/mol. From our *in silico* results, all the investigated Cys could thus theoretically react with activated omeprazole, forming covalent mixed disulphide bonds. The proposed reactivity order is C283>C89>C136. C89 is, however, excluded by the experimental data on CACT mutants. On the basis of these observations, we selected one of the generated solutions for CACT covalently bound to omeprazole on C283, and we docked it with omeprazole. We obtained a set of 23 energetically favored (negative) solutions, with a top docking-score of −6.64 kcal/mol for the non-covalently bound omeprazole molecule. Through this procedure, we demonstrated the possibility that CACT could bind at the same time two omeprazole molecules: one covalently bound to C283 and another non-covalently bound inside the CACT central cavity. This solution is depicted in Fig. 7A.

## Discussion

In the present study, the proteoliposome system reconstituted with the recombinant mitochondrial CACT has been used for investigating the inhibition of the transporter by omeprazole. The compound forms S-S mixed disulphide(s) with critical Cys residues or week interactions with the substrate binding site, which are observed as irreversible or competitive inhibition, respectively. The WT transporter contains thiol groups of Cys exposed towards the extraliposomal environment, which corresponds to the cytosolic side of the transporter [9]. In this context the pharmacological agent after activation by acidic pH as it occurs physiologically in the gastric environment, could react covalently with these Cys residues [14]–[16], [18], [31]. C283 and C136 have been identified as targets of omeprazole, on the basis of data obtained with mutant proteins. The two Cys residues are exposed in the central cavity of the transporter, which face the extra-mitochondrial environment. C136 is in the bottom of the cavity just below the substrate binding site and was previously found to be the main target of small SH reagents such as NEM, which inactivate the transporters; while C283 is located in the upper part of the cavity, far from the carnitine binding site and is not involved in the inhibition by SH reagents [9], [20]. Omeprazole which is much larger than NEM inhibits the transport upon binding to C283 causing steric hindrance and interferences with the translocation process. The relatively high affinity of the transporter for omeprazole (IC50 5.4 µM) should rely on the covalent bond formation and also on several week interactions of hydrophobic and aromatic groups of the compound with hydrophobic residues of the CACT which are located in the neighborhood of C283, as previously described [11]. The double mechanism of interaction is also confirmed by the computational analysis, which predicts the binding of omeprazole in the central cavity of the protein (Fig. 7A). At an upper, more external level, the covalent binding with C283 is predicted for an omeprazole molecule. At a lower level the binding of omeprazole in a non covalent mode is also predicted. This location, which overlaps the substrate binding site, is in agreement with the competitive interaction observed in the C-less protein. Indeed, the computational analysis revealed that the interaction of omeprazole with the transporter involves residues which were previously found to constitute the substrate binding site of carnitine [30]. The possible formation of a disulphide with C136, placed just below the substrate binding site, is experimentally demonstrated and fits very well with the positioning of omeprazole in the lower level. Both methodologies concur to indicate that omeprazole binds to C136 with lower efficiency respect to C283 (see Results secion 3.5 and Fig. 4). Indeed, the slightly lower inhibition of the mutant lacking C283 than of the mutant lacking C136, indicates that this second covalent bond is somewhat less efficient than that with C283; this is confirmed by the finding that the mutant Cless-C136, containing the single C136 residue, and the mutant C283S show the same extent of inhibition (Fig. 4). Surprisingly, the covalent interactions of omeprazole with the Cys residues involved in the inhibition are facilitated by the presence of the substrate. This is explained by conformational changes occurring in this moiety of the protein upon substrate binding [11], which improves the accessibility of C283. This also explains why the pre-incubation with omeprazole produces a stronger inhibition compared to co-incubation during transport assay as observed in Fig. 1. Indeed in pre-incubation procedure the proteoliposomes have 15 mM external carnitine which is then removed by Sephadex G-75 chromatography before the transport assay (see Materials and methods). The similarity of some aspects of the inhibition of the mitochondrial transporter with those previously found for the plasma membrane OCTN2 transporter, which however were not described at the molecular level [19], indicates that the substrate binding sites should have some similar properties, in spite of the very different primary structures and affinity for substrates of the two proteins. [11], [32]. These similarities explain also the common interactions among the two transporters and some drugs, such as β-lactam antibiotics, previously described [3], [33]. As it was reported for the OCTN2, the IC50 of CACT for omeprazole is below the plasma concentrations of the compound following the pharmacological administration. To predict the possible in vivo implications of the results described, it should be taken into account that the CACT transporter is essential in most tissues for the process of fatty acid β-oxidation. The defects of the CACT function, known as secondary carnitine deficiencies, are characterized by encephalopathy, respiratory distress, cardiomyopathy, muscle weakness, myopathy, vomiting and others [34]. The inhibition by omeprazole may lead to a mild deficiency-like syndrome. This correlates well with the symptoms like stomach pain, dizziness, difficult breathing, muscle spasm, tightening and cramping, which are described as side effects of omeprazole [see on the WEB: http://dailymed.nlm.nih.gov/dailymed/drugInfo.cfm?id=4761]. Moreover it has been demonstrated that omeprazole can enter the cells in the activated form leading to toxicity at gastric level due to oxidative damage caused by the interaction and oxidation of Cys residues of proteins [31], [35], [36]. The mechanism of interaction with the CACT could contribute to the oxidative damage as recently found in the case of H_2_O_2_ [27].

In the light of the data presented, the described experimental approach may have on the one hand utility for predicting side interaction of pump inhibitor analogues with the CACT and, hence, improve their safety. On the other hand such tools could be useful for searching specific inhibitors of the CACT, which are of interest, for example, as cardioprotective agents, as it was previously described for mildronate which plays benefits under ischemic conditions [2].
